# The Role of miR-342 in Vascular Health. Study in Subclinical Cardiovascular Disease in Mononuclear Cells, Plasma, Inflammatory Cytokines and *PANX2*

**DOI:** 10.3390/ijms21197217

**Published:** 2020-09-29

**Authors:** Sabina L. Ray, David J. Coulson, Megan Li Yuen Yeoh, Alice Tamara, Jevi Septyani Latief, Sherin Bakhashab, Jolanta U. Weaver

**Affiliations:** 1 Translational & Clinical Research Institute, Newcastle University, Newcastle Upon Tyne NE2 4HH, UK; Sabina.Ray39@gmail.com (S.L.R.); davidcoulson97@outlook.com (D.J.C.); myly26@gmail.com (M.L.Y.Y.); Alicelie96@hotmail.com (A.T.); jevi.septyani@gmail.com (J.S.L.); Sherin.Bakhashab2@newcastle.ac.uk (S.B.); 2 Biochemistry Department, King Abdulaziz University, P.O. Box 80218, Jeddah 21589, Saudi Arabia; 3 Department of Diabetes, Queen Elizabeth Hospital, Gateshead, Newcastle Upon Tyne NE9 6SH, UK; 4 Vascular Biology and Medicine Theme, Newcastle University, Newcastle Upon Tyne NE2 4HH, UK

**Keywords:** *CXCR1/2*, *PANX2*, TNF-α, inflammation, miR-342-3p/-5p, therapeutic targets, TIMP-1, T1DM, CVD

## Abstract

Cardiovascular disease (CVD) correlates with inflammation and a reduction in circulating endothelial progenitor cells (cEPCs). Recently, CVD was shown to be the main cause of mortality in individuals with type 1 diabetes (T1DM). In animals, miR-342 was shown to exert an anti-inflammatory effect in CVD. Hypothesis: miR-342-3p/-5p are downregulated in subclinical CVD (T1DM), whereas inflammatory cytokines are upregulated. We studied miR -342 -3p/5p in plasma/peripheral blood mononuclear cells (PBMCs) in 29 T1DM and 20 controls (HC). Vascular health was measured by fibronectin adhesion assay (FAA), cEPCs (CD45^dim^CD34^+^133^+^ cells) and by assessing inflammation and tissue inhibition of metalloproteases (TIMP-1). In T1DM IL-7, IL-8, TNFα and VEGF-C were increased in plasma. MiR-342-3p/-5p were downregulated in PBMCs in T1DM, but not in plasma. *PANX2*, chemokine receptors *CXCR1/2* mRNAs, were increased in PBMCs in T1DM. MiR-342-3p was negatively correlated with TIMP-1, IL-6, IL-8, TNF-α, HbA_1c_ and *CXCR2*, whilst miR-342-5p was negatively correlated with TIMP-1, IL-6, IL-8 and HbA_1c_. There was a positive correlation among miR-342-3p, FAA and cEPCs, and between miR-342-5p and cEPCs. ROC curve analyses showed significant downregulation of miR-342-3p/-5p at HbA_1c_ > 46.45 mmol/mol, indicating their potential as biomarkers for subclinical CVD. Our findings validated animal studies and confirmed the proangiogenic properties of miR-342-3p/-5p. MiR-342-3p/-5p-based intervention or monitoring may prove to be beneficial in managing CVD.

## 1. Introduction

Inflammatory processes have been shown to be key for the development and complications of cardiovascular disease (CVD) [1]. CVD is currently the predominant cause of early mortality in type 1 diabetes patients (T1DM). Data has suggested life expectancy decreases by approximately 13 years in T1DM [2].

We have previously published evidence to suggest that T1DM has features of early CVD without documented or apparent CVD events [3,4,5]. Patients with T1DM were characterized by a significant endothelial dysfunction, inflammatory state, reduced circulating endothelial progenitor cells (cEPCs), pro-angiogenic cells (PACs) and colony forming units (Hills). All of those vascular health indicators have been noted to characterize CVD in other patient groups [6,7]. Although evidence suggests CVD is strongly correlated and predicted by the markers of vascular health listed above, other biomarkers are becoming apparent, including microRNAs (miRNA) [8].

MiRNAs are highly conserved, noncoding RNA molecules of approximately 22 nucleotides in length that exert posttranscriptional effects on gene expression [9]. They are expressed in a tissue- and cell-type specific manner, and play essential roles in many biological processes, including inflammation. Since the discovery of miRNAs in 1993, they have been established as novel biomarkers for many conditions, including CVD [10]. This group of gene expression regulators have also been described in type 1 diabetes mellitus (T1DM) patients [11], and there are many miRNAs that have been shown to have potential to act as biomarkers for T1DM [12]. However, understanding of the specific roles of miRNA in this disease is lacking.

Currently, there are strong links between miRNA and their role in many cellular pathways, including cell proliferation, apoptosis and immune response pathways, to name a few [13]. This introduces the idea that miRNAs may be responsible for the regulation of inflammation associated with T1DM and the association between their regulation and cardiovascular health. Previous evidence has demonstrated a strong correlation between T1DM and increased risk of CVD [14]. If miRNAs could modulate inflammatory cytokine concentration and cell adhesion, for example, this, in turn, could have a positive impact on cardiovascular health and CVD risk in T1DM patients.

Out of 2000 miRNAs that have been discovered in humans, miR-342-3p/-5p appear to have great potential to repress inflammation in atherosclerosis [15,16]. Additionally, current studies have shown miR-342-3p l to increase the cell survival, motility and proliferation of osteoclast precursors [17].

Takahashi et al. observed that some microRNAs were consistently dysregulated in T1DM and involved in the development of autoimmunity and inflammation [18]. MiR-342 was listed as one of these microRNAs which was observed to be downregulated in T regulatory cells of T1DM patients. Furthermore, Hezova et al., 2010, found miR-342 to be downregulated in T regulatory cells in T1DM patients, further indicating that this miR could be involved in developing autoimmunity and increased inflammation in T1DM patients [19]. MiR-342-3p specifically has also been described to be associated with obesity in mice and, therefore, the immune response, providing further links between this miR and CVD in T1D [20]. Moreover, since T1DM displays features of endothelial dysfunction before CVD occurs, it is classed as subclinical CVD [21].

To our knowledge, no studies have been carried out in patients with T1DM and vascular health or CVD. If we understood the mechanism behind the action of miR342 in vascular health, this could facilitate further research into new CVD therapies. We thus hypothesize that miR-342-3p/-5p are downregulated in T1DM patients and may be associated with a deterioration of vascular health.

## 2. Results

### 2.1. Patient Phenotypes

Subjects with type 1 diabetes were relatively well controlled (HbA_1c_ 57.3 ± 7.6 mmol/mol), with a duration of diabetes of 22.4 ± 3.9 years and no documented CVD events. Patients were age- and sex-matched with healthy controls (HC, Table 1).

### 2.2. MicroRNA-342 Isoform Expression

**Figure 1:**
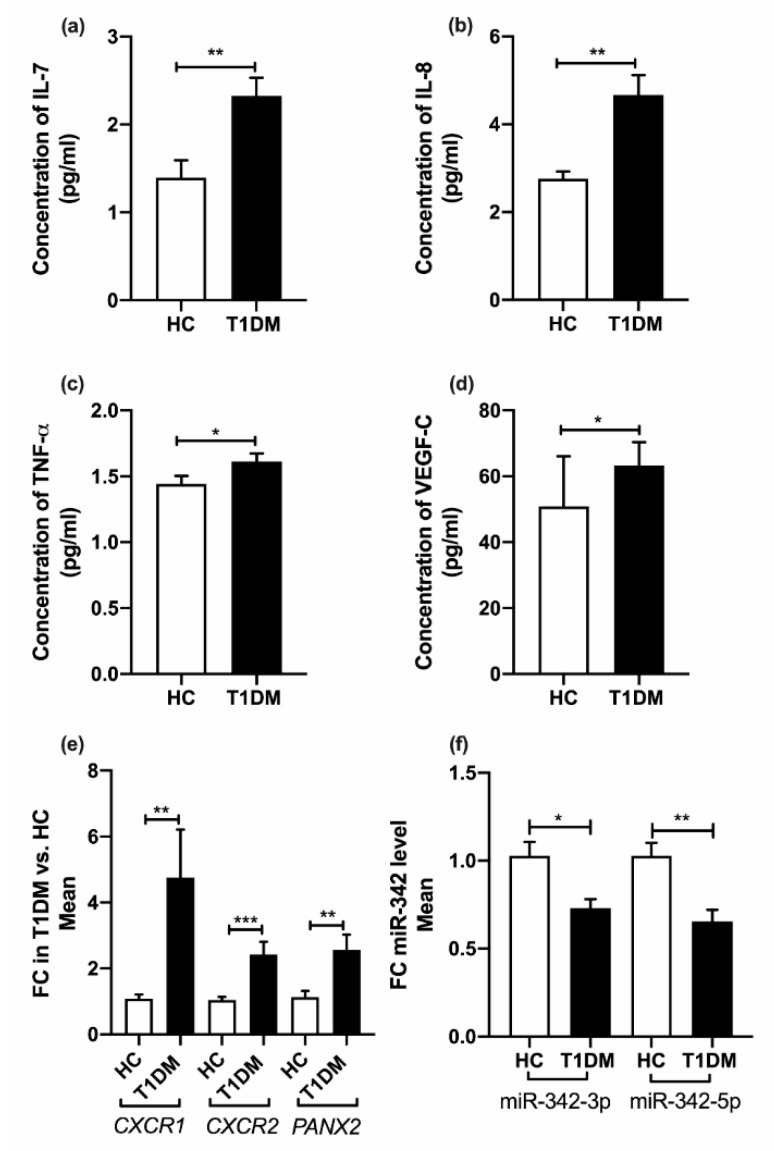
Comparison of data between patients with type 1 diabetes and healthy controls. (**a**) Levels of IL-7 in plasma, (**b**) levels of IL-8 in plasma, (**c**) levels of TNF-α in plasma, (**d**) levels of VEGF-C in plasma (**e**) mRNA expression of *CXCR1*, *CXCR2* and *PANX2* in PBMCs (**f**) miR-342-3p and miR-342-5p expression in PBMCs. Data are presented as mean ± SE, analyzed by unpaired *t*-tests or Mann-Whitney U test accordingly. FC—fold change; HC—healthy control; T1DM—type 1 diabetes mellitus. * *p* < 0.05, ** *p*< 0.01 *** *p*< 0.001.

Both miR-342-3p/-5p were significantly downregulated in T1DM patients compared to HC; fold change (FC) = −1.4, *p* = 0.01; FC = −1.6, *p* = 0.002 respectively in peripheral blood mononuclear cells (PBMCs), but not in plasma (Figure 1f).

### 2.3. Cytokine Profiles

Pro-inflammatory cytokines IL-8 and TNF-α, homeostatic cytokine IL-7 and growth factor VEGF-C were increased in patients’ plasma with T1DM (4.7 ± 1.3 pg/mL, 1.6 ± 0.2 pg/mL, 2.3 ± 0.6 pg/mL and 63.2 ± 20.3 pg/mL) versus HCs (2.8 ± 0.5 pg/mL, 1.4 ± 0.2 pg/mL, 1.4 ± 0.6 pg/mL, 50.8 ± 48.2 pg/mL respectively); *p* = 0.003, *p* = 0.041, *p* = 0.008 and *p* = 0.013 respectively (Figure 1a–d). No significant changes were observed in IL-6 and TIMP1 levels in patients (0.60 ± 0.42 pg/mL, and 5.3 ± 0.11 pg/mL) compared to HCs (0.43 ± 0.28 pg/mL, and 5.2 ± 0.06 pg/mL respectively). In PBMCs, *CXCR1* and *CXCR2* mRNA were significantly upregulated in T1DM compared to HCs; FC = 4.3, *p* = 0.009 and FC = 2.3, *p* < 0.001 respectively (Figure 1e). The CXCR1:CXCR2 mRNA ratio was similar (1.589:1 in T1DM and 1.556:1 in HCs).

Moreover, we previously reported that the indices of vascular health, i.e., cEPC/CD45^dim^CD34^+^CD133^+^ cells, PACs, and fibronectin adhesion assay (FAA), were lower in T1DM patients versus HC [4].

### 2.4. Pannexin-2 (PANX2) (Target of miR-342-3p) mRNA Expression

In PBMCs, the miR 342 target gene *PANX2* mRNA was significantly upregulated in T1DM versus HCs; FC = 2.26, *p* = 0.006 (Figure 1e).

### 2.5. Association between miR-342 Isoforms and Inflammatory Markers

**Figure 2:**
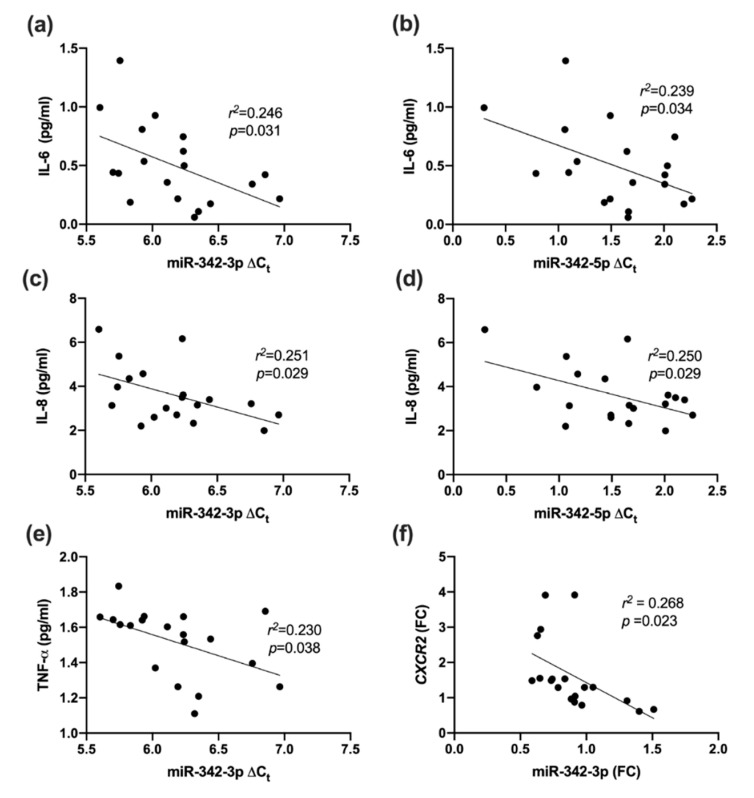
Correlation between miR-342-3p/-5p and inflammatory markers in type 1 diabetes and healthy controls combined. (**a**) miR-342-3p and IL-6; (**b**) miR-342-5p and IL-6; (**c**) miR-342-3p and IL-8; (**d**) miR-342-5p and IL-8; (**e**) miR-342-3p and TNF-α; (**f**) miR-342-3p and chemokine receptor type 2 (*CXCR2)*. FC—fold change.

In all studied subjects (T1DM and HCs), miR-342-3p was inversely correlated with IL-6 (*r*^2^ = 0.246, *p* = 0.031), IL-8 (*r*^2^ = 0.251, *p* = 0.029) and TNF-α (*r*^2^ = 0.230, *p* = 0.038). There was also a negative correlation between miR-342-5p and IL-6 (*r*^2^ = 0.239, *p* = 0.034) and IL-8 (*r*^2^ = 0.250, *p* = 0.029, Figure 2a–e).

### 2.6. Association between miR-342 Isoforms and Inflammatory Receptors

miR-342-3p was negatively correlated with *CXCR2*; *r*^2^ = 0.268, *p* = 0.023 (Figure 2f), but not with *CXCR1*. In contrast, miR-342-5p was not significantly correlated with *CXCR1/2*.

### 2.7. Association between miR-342 Isoforms and Vascular Health (Fibronectin Adhesion Assay, CD45^dim^CD34^+^133^+^ Cells and TIMP-1)

**Figure 3:**
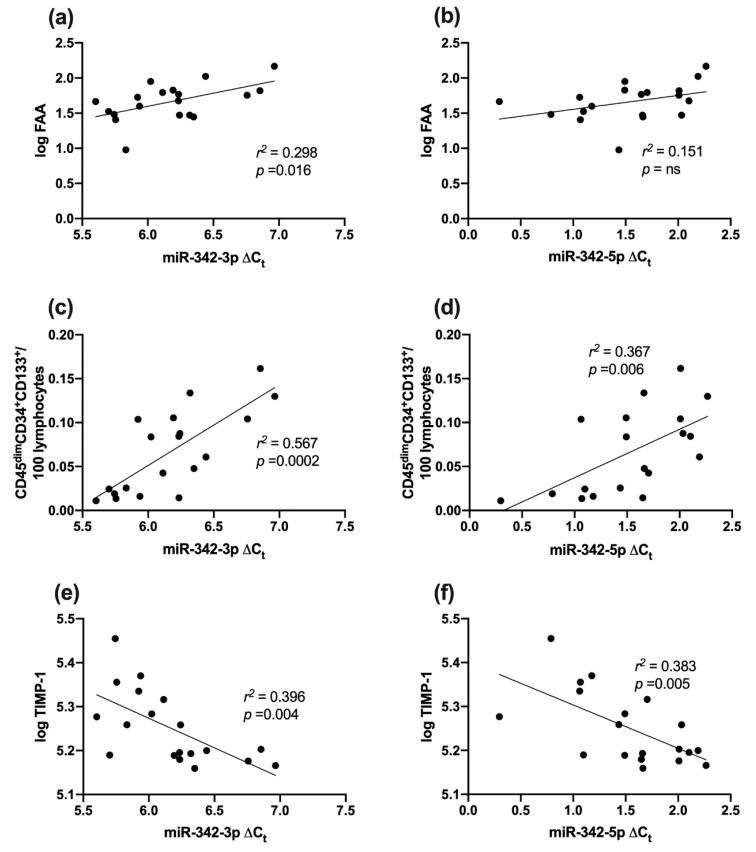
Correlation between miR-342-3p/-5p expression in PBMCs and vascular health in type 1 diabetes and healthy controls combined. (**a**) miR-342-3p and log FAA; (**b**) miR-342-5p and log FAA; (**c**) miR-342-3p and CD45^dim^CD34^+^CD133^+^; (**d**) miR-342-5p and CD45^dim^CD34^+^CD133^+^; (**e**) miR-342-3p and log TIMP1 (**f**) miR-342-5p and log TIMP1. FAA—fibronectin adhesion assay; ns—nonsignificant; TIMP-1—tissue inhibitor of metalloproteinases 1.

MiR-342-3p/-5p isoforms were both positively correlated with FAA; *r*^2^ = 0.291, *p* = 0.016; *r*^2^ = 0.151, *p* = 0.099 (nonsignificant) respectively (Figure 3a,b). Moreover, miR-342-3p/-5p were positively correlated with endothelial progenitor cells, CD45^dim^CD34^+^CD133^+^; *r*^2^ = 0.567, *p* = 0.0002, *r*^2^ = 0.367, *p* = 0.006 respectively (Figure 3c,d). In contrast, both isoforms were inversely correlated with log TIMP-1; *r*^2^ = 0.396, *p* = 0.004, *r*^2^ = 0.383, *p* = 0.005 respectively (Figure 3e,f).

### 2.8. Correlation between miR-342 Isoforms and HbA1c

**Figure 4:**
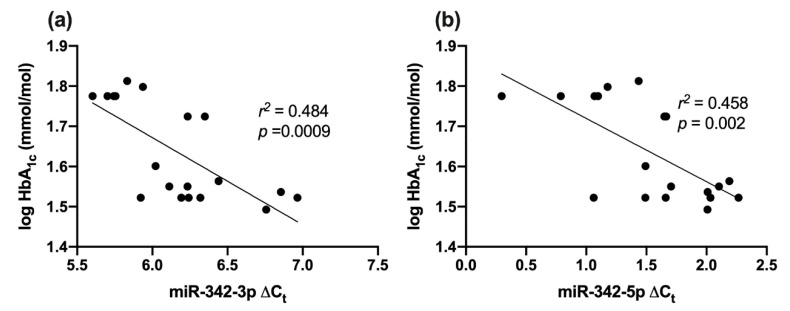
Correlation between miR-342-3p/-5p and glycemic control in type 1 diabetes and healthy controls combined. (**a**) miR-342-3p and log HbA_1c_; (**b**) miR-342-5p and log HbA_1c_.

Both miR-342-3p and -5p were negatively correlated with log HbA_1c_; *r*^2^ = 0.484, *p* = 0.0009; *r*^2^ = 0.458, *p* = 0.002 respectively (Figure 4a,b).

### 2.9. Receiver Operating Characteristic Curve Analysis for miR342-3p and 342-5p

**Figure 5:**
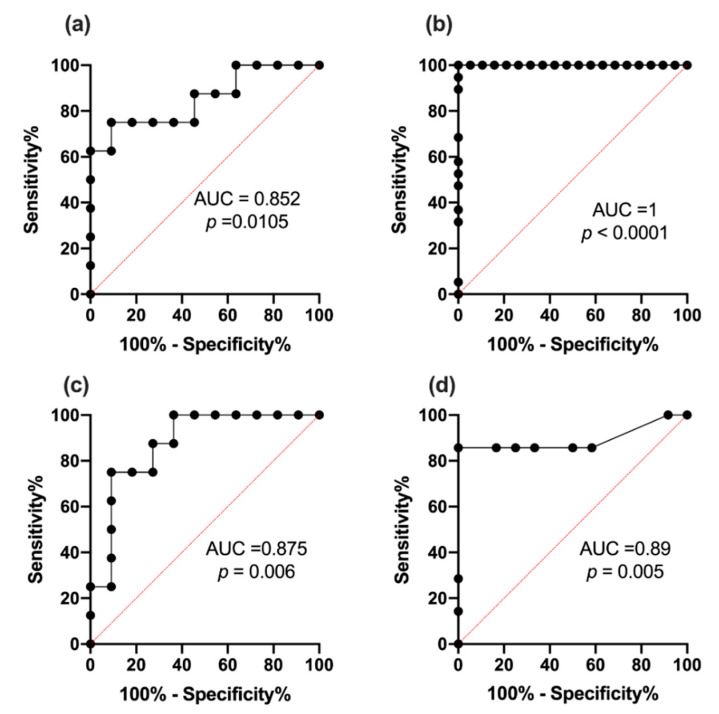
(**a**) ROC curve for miR-342-3p in type 1 diabetes and healthy controls; (**b**) ROC curve of HbA_1c_ indicating downregulated miR-342-3p expression; (**c**) ROC curve for miR-342-5p in type 1 diabetes and healthy controls; (**d**) ROC curve of HbA_1c_ indicating downregulated miR-342-5p expression.

Two receiver operating characteristic curve analyses (ROC) were performed for each miR studied. ROC analyses showed (1) that miR-342-3p was able to distinguish between T1DM and HC (*p* = 0.0105; AUC 0.852) with a sensitivity of 75% and 91% specificity (Figure 5a), (2) significant downregulation of miR-342-3p (*p* < 0.0001, AUC 1) with a sensitivity of 100% and 85% specificity defined subclinical CVD at HbA_1c_ > 46.45 mmol/mol, (6.4%), *p* < 0.0001 (Figure 5b).

ROC curve analyses showed (1) that miR-342-5p-5p was able to distinguish between T1DM and HC (*p* = 0.006; AUC 0.875) with a sensitivity of 75% and 91% specificity (Figure 5c), (2) significant downregulation of miR-342-5p (*p* = 0.005; AUC 0.89) with a sensitivity of 86% and 83% specificity defined subclinical CVD at HbA_1c_ > 46.45 mmol/mol, (6.4%), *p* = 0.005 (Figure 5d).

## 3. Discussion

In the current research, we validate animal findings on miR-342-3p/-5p. Furthermore, we confirm the presence of inflammation in otherwise healthy patients within T1DM by showing increased plasma concentrations of inflammatory, TNF-α, IL-6 and IL-8, as well as IL-7 homeostatic/inflammatory cytokine. In addition, we report, for the first-time, the downregulation of isoforms miR-342-3p/-5p in PBMCs, but not in plasma, in patients with T1DM compared to HC. Moreover, to our knowledge, we are the first to demonstrate a negative correlation between miR-342-3p/-5p and glycemic control_,_ TIMP-1, TNF-α and pro-inflammatory cytokine receptor *CXCR2*, as well as positive correlations between miR-342-3p/-5p and vascular health; FAA and CD45^dim^CD34^+^CD133^+^ cells, otherwise known as early endothelial progenitor cells.

### 3.1. Inflammatory Markers in T1DM

Our results showed an increase in IL-7 in T1DM, which implies that IL-7 plays a role in inflammation in T1DM; this is concordant with other studies. A previous study showed that IL-7 enhances the expansion of CD4^+^ and CD8^+^ T cells in an autoimmune diabetes mice model [22]. IL-7 was also shown to be involved in the generation and maintenance of T-cell autoimmunity in T1DM [23] and to provide essential signals for the generation of human memory stem T cells from naive precursors [24]. Furthermore, through blocking the receptor IL-7R, we were able not only to prevent, but also reverse, the onset of diabetes in mice [25].

IL-8 was also shown to be increased in recent, in accordance with others and our previous research [5,26]. This cytokine plays a role in activating inflammatory cells, including neutrophils, and is modulated by the differential activation of CXCR1/2 [27]. In line with our results, we observed increased *CXCR1* and *CXCR2* levels in PBMC in T1DM. The upregulated *CXCR1* and *CXCR2* mRNA in PBMCs upon elevation of IL-8 concentration acts as a positive feedback system to provide continuous stimulation of the inflammatory pathway.

In addition to IL-7 and IL-8 being raised in our subjects, another pro-inflammatory cytokine, TNF-α, was also increased. TNF-α is a well-established cytokine involved in the inflammatory response. It presents during inflammation and is reported to affect other inflammatory markers [28]. Thus, our results on IL-7, IL-8, *CXCR1*, *CXCR2* and TNF-α confirm T1DM as an inflammatory disease.

### 3.2. MiR-342-3p Expression in PBMC in T1DM

We are the first to report the downregulation of both isoforms of miR-342-3p and -5p in PBMCs but not plasma in T1DM. Although previous studies have described a decreased expression of miR-342 in regulatory T cells compared with T cells in T1DM, no information is presently available on isoforms miR-342-3p or 5p [19].

In T1DM patients, the downregulation of miR-342-3p/-5p may account for the increased concentration of inflammatory cytokines. This was investigated in another study on atherosclerosis, in which a reduction of inflammation was observed following the inhibition of nuclear enriched abundant transcript 1 (NEAT1) and subsequent overexpression of miR-342-3p [16]. The overexpression of miR-342-3p in response to the inhibition of NEAT1 inhibited the release of inflammatory cytokines IL-6, IL-1β, TNF-α and cyclooxygenase-2 (COX-2). This strongly supports our finding in patients, and can be applied to the study of the causal relationship of miR-342-3p downregulating inflammatory cytokines in our study. 

**Figure 6:**
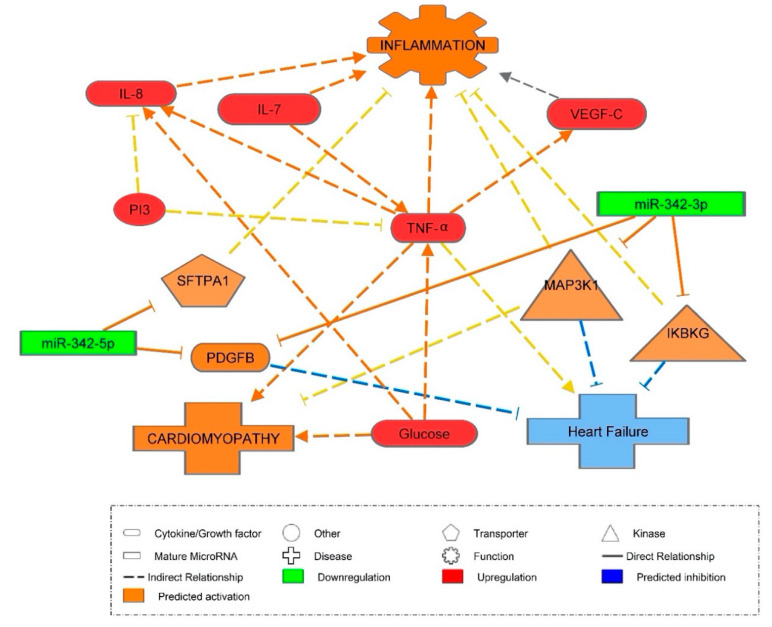
Ingenuity Pathway Analysis (IPA) prediction network of miR-342-3p/-5p and cardiovascular disease. Downregulation is shown in green and upregulation in red. Orange signifies predicted activation of a molecule or biological function. Paler orange signifies a prediction of less activation. Blue signifies the inhibition of a molecule or biological function. Orange lines represent stimulation, and blue lines inhibition and grey lines reciprocal stimulation and inhibition. Solid uninterrupted lines represent a direct action and dashed interrupted lines represent an indirect action. It has been predicted, through animal knockout studies, that miR-342-5p will activate surfactant protein A1 (SFTPA1) and platelet derived growth factor subunit B (PDGFB) which, in turn, will inhibit heart failure and inflammation. miR-342-3p activates inhibitor of nuclear factor kappa B kinase regulatory subunit gamma (IKBKG), mitogen activated 3 kinase 1 (MAP3K1) and also PDGFB, which are responsible for inhibiting heart failure and inflammation. It is predicted that D-glucose will activate cardiomyopathy, TNF-α and IL-8, and that IL-8 will activate inflammation whilst TNF-α activates VEGF-C as well as activating inflammation. As some studies have suggested otherwise regarding VEGF-C activating inflammation, this arrow appears in grey. It is predicted that IL-7 will activate TNF-α, resulting in activation of inflammation, whilst phosphoinositide 3-kinase (PI3), when activated, is predicted to inhibit TNF-α and IL-8.

Furthermore, after inputting miR-342-3p, miR-342-5p, IL-7, IL-8, TNF-α, VEGF-C and glucose into IPA software simulating a diabetic state, it was predicted, through published knockout studies, that miR-342-3p had anti-inflammatory effects. miR-342-3p was expected to activate mitogen-activated protein 3 kinase 1 (MAP3K1), inhibitor of nuclear factor kappa B kinase regulatory subunit gamma (IKBKγ), and platelet derived growth factor subunit B (PDGFB) (Figure 6). These kinases and growth factors indirectly inhibit inflammation, heart failure and cardiomyopathy. IPA predicted these causal relationships from knockout studies such as IKBKγ gene knockout, resulting in increased inflammation of liver, etc. Similarly, IPA also predicted that IL-7, IL-8 and TNF-α would activate the inflammatory response, as well as confirming the hypothesis that elevated glucose concentrations caused increased TNF-α and IL-8 expression.

### 3.3. MiR-342-5p Expression in PBMCs in T1DM

Similar to miR-342-3p, miR-342-5p was also downregulated in T1DM patients. Both correlated with similar molecules; however, miR-342-5p was not correlated with *CXCR2* mRNA or TNF-α. This indicates that miR-342-5p may regulate similar pathways to miR-342-3p, but that miR-342-3p may be more pro-angiogenic than miR-342-5p.

After generating predicted targets using IPA, there is some uncertainty as to whether miR-342-5p is pro-inflammatory or anti-inflammatory. This is because one study has shown miR-342-5p to be pro-inflammatory in the late stages of atherosclerosis [29]. However, IPA also predicted miR-342-5p to inhibit inflammation through targeting surfactant protein A1 (SFTPA1). These data was born out of experiments in mutant mouse SFTPA1 knockout resulting in increased lung inflammation. Several other causations showed SFTPA1 to be anti-inflammatory through gene knockouts in mice supporting miR-342-5p is anti-inflammatory. Hence, the prediction shown in Figure 6 demonstrates that miR-342-5p inhibits the inflammatory response through the activation of SFTPA1 and PDGFB.

Interestingly, PDGFB was activated by both miR-342-3p/-5p, further suggesting that both may regulate similar pathways. PDGFB has been shown to inhibit heart failure through a mutant mouse PDGFB gene knockout, resulting in an increased congestive heart failure. As T1DM has been found to be linked to CVD, this prediction of inhibition of heart failure through miR-342-3p/-5p may prove to be beneficial to T1DM patients.

### 3.4. Inflammatory Marker Associations with miR-342

We have shown there were negative correlations between miR-342-3p and IL-6, and IL-8 and TNF-α. Interestingly, miR-342-5p showed negative correlation with IL-6 and IL-8; however, unlike miR-342-3p, this was not the case with TNF-α. Therefore, as miR-342-3p/-5p increased, these pro-inflammatory cytokines were shown to be downregulated, confirming our findings that miR-342-3p/-5p have potential anti-inflammatory effects. Other studies support our conclusion that miR-342-3p is protective against atherosclerosis, as when miR-342-3p was downregulated in endothelial cells in mice, an increase in chitinase 3 like 1 (Chi3l1), a mediator of endothelial inflammation, was observed [15]. This resulted in subsequent vascular inflammation.

In contrast to this, in a state of established atherosclerosis (atherosclerotic plaques), miR-342-5p has been described to induce pro-inflammatory mediators including nitric oxide synthase 2 (Nos2) and IL-6 [30]. This is in contrast to the observations made in our study, as it was performed in PBMCs from T1DM, and as such, it is understandable that the results would differ and that miR-342-5p may exert different effects at different stages of inflammatory diseases. Reviewing miR-342-5p expression in established atherosclerosis is likely to be complex, as many other factors may be involved in the late stages of the atherosclerotic process. 

A negative correlation was also observed between miR-342-3p and cytokine receptors *CXCR2* mRNA. The latter, being the receptor of IL-8, CXCR1 and CXCR2, are responsible for trafficking inflammatory mediators [27]. This negative correlation provides further evidence that miR-342-3p/-5p may play a role in regulating the inflammatory response. As inflammation in T1DM has been found to be associated with an increased risk of CVD, regulating inflammation may decrease this risk.

### 3.5. Vascular Health and miR-342

We found positive correlations between miR-342-3p/5p with FAA. Cell adhesions are crucial for cell regeneration, motility and angiogenesis [3,31]. This suggests that the greater the miRNA concentration, the greater the cell adhesion. This association can be converted into a causal relationship, since in a recent study, the upregulation of miR-342-3p was shown to lead to the promotion of cell survival and motility [17].

In addition, we reported that miR-342-3p/-5p were positively correlated with CD45^dim^CD34^+^CD133^+^ cells. otherwise known as circulating early endothelial progenitor cells (EPCs). It is well established, including by us, that in both type 1 and type 2 diabetic patients, there are fewer circulating EPCs than in HCs, confirming increased CVD risk [4,32]. EPCs have been proven to promote vascular repair via transmigration to promote new vessel growth and predict future CVD events [6,33]. It is well known that almost all risk factors for CVD cause dysfunction or a decrease of EPCs, validating our findings [34].

In line with our hypothesis, we established a negative correlation between miR-342-3p/-5p and TIMP-1. As TIMP-1 is a matrix metalloproteinases (MMP) inhibitor, decreasing TIMP-1 would lead to an increase in cell motility, and hence, increased vascular repair [35]. Transendothelial migration is dependent upon the degradation of basement membranes. This degradation process relies on the production of enzymes which are capable of degrading the collagenous membranes, such as MMPs. TIMP-1 is an inhibitor of many MMPs, such as MMP-9, and therefore, negative correlation with the microRNA family miR-342 may suggest that TIMP-1 regulation is dependent on miR-342-3p/-5p [36].

### 3.6. miR-342 Affecting Hyperglycemia

We found an inverse correlation between miR-342-3p/-5p and logHbA_1c_. This is concordant with the findings in an expression study where hyperglycemia was shown to reduce the expression of miR-342-3p and to block vasculogenesis [37]. To our knowledge, no study has examined miR-342-3p/-5p in relation to HbA_1c_. Although our patients were relatively well controlled, the negative relation between glycemic control across all subjects and miRNA-342-3p/-5p confirmed the proangiogenic properties of this miR.

### 3.7. ROC Analysis

Our data showed that the defining point of our miR downregulation (associated with T1DM) defined subclinical CVD, and that this occurred at HbA_1c_ > 46.45 mmol/mol, (6.4%). We can thus postulate that miR-342-3p/-5p is a biomarker for early subclinical CVD, as the difference in miR-342-3p/-5p expression could be attributed to the onset of hyperglycemia. It is of interest that the value of HbA_1c_ achieved from ROC curve analysis, i.e., 46.5 mmol/mol (6.4%), also signifies the turning point for the development of diabetes and is commonly used to describe a prediabetes state associated with the development of microvascular complication [38,39].

In this study of well characterized controls and diabetic patients, we independently found, by using miR-342-3p/-5p, the biochemical set point for diabetic complications. This cutoff defined an increased CVD risk and appeared to coincide within the prediabetes range (42–47 mmol/mol or 6.0–6.49%). Although this is a small study, this independent analysis could be used to confirm that miR342-3p/5p is a biomarker for the beginning of subclinical CVD.

This finding has clinical relevance, as reliable diagnoses of subclinical CVD are notoriously difficult without using invasive methods. Therefore, we hypothesize that miR-342-3p/-5p may act as either a biomarker of CVD onset or of CVD progression, or both. The downregulation of miR-342-3p/-5p emphasizes increased cardiovascular risk.

### 3.8. Pannexin-2 mRNA Target of miR-342-3p

**Figure 7:**
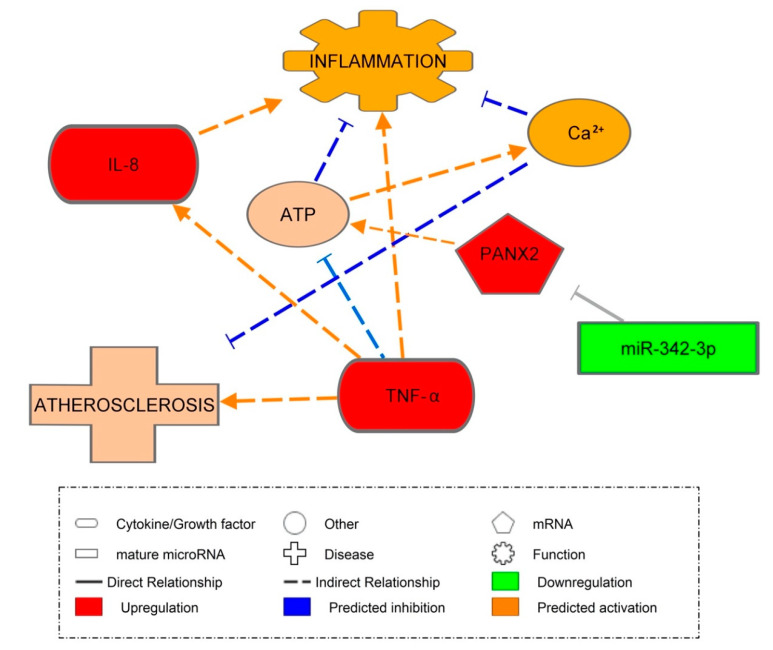
MiR-342-3p and inflammation. IPA prediction network representing the activation of PANX2 by miR-342-3p and its effect on inflammatory response. Orange signifies the predicted activation of a molecule or biological function. Paler orange signifies a prediction of less activation. Blue signifies the inhibition of a molecule or biological function. Orange lines represent stimulation and blue lines inhibition. Solid uninterrupted lines represent a direct action and dashed interrupted lines represent an indirect action. It was predicted that miR-342-3p would inhibit PANX2. However, when PANX2 was activated, it activated ATP, which was predicted to be responsible for indirectly activating calcium (Ca^2+^) release to inhibit inflammation. It was also predicted that ATP itself would indirectly inhibit inflammation, and that TNF-α would activate the process of atherosclerosis indirectly, and also activate IL-8, which, in turn, activates inflammation.

Through inputting our significant mRNAs into a miR-gene target filter in IPA (Figure 7), it was predicted that *PANX2* would be the mRNA target for miR-342-3p. We found *PANX2* to be increased in PBMCs and predicted them to be activated by miR-342-3p. *PANX2* is a member of the newly discovered 3-member family of proteins expressed in brain and tissue. It was predicted that the upregulation of *PANX2* would indirectly result in the increased release of ATP. ATP has many functions in different pathways and may be pro- or anti-inflammatory. However, IPA generated inhibitory causation between ATP and inflammation in response to miR-342-3p involvement [40]. IPA also predicted a link between an influx of calcium and the influx of ATP, which led to an indirect anti-inflammatory response. Other studies have indicated that *PANX2* might be involved in providing a flux pathway for ions such as ATP and Calcium [41]. Berchtold et al. found that the expression of *PANX2* is downregulated by pro-inflammatory cytokines in rat islets and INS-1E cells [42]. Bond and Naus further supported the involvement of *PANX2* in inflammation as well as ATP signaling; their absence has been explicitly verified in macrophages [43].

The results from both our study and others, including that of Wang et al., suggest that the downregulation of miR-342-3p could contribute to one of the world’s most prevalent diseases, CVD [16]. As CVD has been proven to be accelerated by T1DM, these results help support the idea that miR-342-3p could form the basis of therapeutic interventions and the monitoring of CVD. [44]. Our cross-sectional findings need to be further validated in prospective longitudinal studies on the progression of CVD.

## 4. Materials and Methods

We recruited 29 patients with T1DM with inclusion criteria of HbA_1c_ < 8.5% (69 mmol/mmol), absence of macrovascular disease or stage 3b renal impairment (eGFR < 45 mL/min/1.73 m^2^) or active proliferative retinopathy. Patients were matched with 20 age and gender-matched, nondiabetic HC. Patients with T1DM were recruited either from Queen Elizabeth Hospital, Gateshead or Royal Victoria Infirmary, Newcastle, UK. All subjects gave informed consent and the study was performed in accordance with the Helsinki Declaration. The study was approved by the NHS Health Research Authority, NRES Committee North East-Sunderland, UK (Research Ethics Committee Reference Number 12/NE/0044).

Power calculation was undertaken as part of a previously published study to detect improvement in EPCs [13]. The minimum number of patients/healthy controls required was 20 in each group. The data from baseline from that study were used to compare T1DM patients and HCs in this substudy. Routine laboratory investigations (full blood count, U&Es, liver function test, thyroid function test and HbA_1c_), 12-lead ECG, blood pressure, weight, height and BMI measurements were performed. 

### 4.1. Meso Scale Discovery (MSD) Assay

Plasma samples from patients and the control group were assayed using K15050D V-PLEX Cytokine Panel 1 human kit, K15049D V-PLEX Proinflammatory Panel 1 human kit, K15190D V-PLEX Angiogenesis Panel 1 human kit and K151JFC human TIMP-1 kit (Meso Scale Discovery, Rockville, MD, USA) in accordance with the manufacturer’s protocol. Plates were read with MSD Sector Imager 2400, and data were analyzed using the MSD Discovery Workbench v. 2.0 software.

### 4.2. Flow Cytometric Evaluation of Circulating Endothelial Progenitor Cells

A flow cytometry on BD FACS Canto™ II system was used previously by us to determine cEPCs/CD45^dim^CD34^+^ CD133^+^ [4].

### 4.3. Fibronectin Adhesion Assay (FAA)

This assay was carried out as previously described [4].

### 4.4. Extraction of MicroRNAs from Plasma

In order to extract microRNAs from plasma, blood samples were centrifuged for 15 min at 500× *g* to separate platelet-poor plasma. The platelet-poor plasma was extracted and centrifuged for a further 5 min at 13,000× *g*. This plasma was stored at −80 °C for further analysis. During this analysis, the samples were tested for hemolysis. MicroRNA was extracted from the centrifuged plasma with a proprietary RNA isolation protocol optimized for serum/plasma by QIAGEN (Exiqon Services, Vedbæk, Denmark). After extraction, the integrity of RNA samples was assessed using Agilent 2100 (Santa Clara, CA, USA) yielding RNA Integrity Numbers (RIN) between 9.1–10 (high).

### 4.5. Extraction of MicroRNA and mRNA from PBMCs

To extract microRNAs and mRNAs from PBMC, peripheral blood was collected and PBMCs were isolated through Ficol separation after an overnight fast. After isolation, cells were lysed with Trizol lysis buffer and lysates were collected and stored at −80 °C for analysis. Total RNA was extracted from PBMCs using miRNEasy Kit (QIAGEN, Hilden, Germany). After extraction, the integrity of RNA samples was assessed using Agilent 2100 (Santa Clara, CA, USA) yielding RIN between 9.1–10 (high).

### 4.6. Assay of miRNA and mRNA Using Real-Time Quantitative PCR

miRNAs were assayed in plasma and PBMCs with the miRCURY LNA RT Kit (QIAGEN, Hilden, Germany). First, 10 ng RNA was reverse transcribed, resulting in cDNA which was diluted 100x and assayed in 10 μL PCR reactions in compliance with the procedure for miRCURY LNA miRNA PCR. Using qPCR on the microRNA Ready-To-Use PCR, each microRNA was assayed once with Pick and Mix using miRCURY LNA SYBR Green master mix. Both positive and negative controls from the reverse transcription reaction were performed and profiled similarly to the samples. Amplification was performed in a LightCycler^®^ 480 Real-Time PCR System (Roche, Basel, Switzerland) in 384 well plates. Using the Roche LC software, amplification curves were analyzed to determine *ΔCt* values. Equation (1) was then used to calculate *ΔΔCt* values, and Equation (2) to calculate fold change (see below for Equations (1) and (2)).

(1) ΔΔCt=(ΔCt×T1DM)−(ΔCt×HCs)

(2) Fold Change=2×|ΔΔCt|

IPA software v. 9 (Ingenuity, Redwood City, CA, USA) aided in the identification of target genes, cellular functions and pathological states regulated by miR-342-3p or -5p.

### 4.7. Statistical Analysis

Data were presented as mean ± SEM, unless stated otherwise. The normality of the data was assessed by Shapiro-Wilk tests, and data were log transformed as appropriate. Comparisons between the two study groups were analyzed using unpaired t-test or Mann Whitney U test. Associations were examined by a linear regression test. A ROC curve analysis of miR-342-3p/-5p was used to assess the sensitivity of miR-342-3p/-5p as a biomarker for subclinical CVD. Moreover, a ROC curve analysis of significantly decreased miR-342-3p/-5p and HbA_1c_ was performed to determine the cut-off value for miR-342-3p/-5p downregulation. All statistical analyses were performed using IBM SPSS Statistics software version 25 (SPSSTM Inc., Armonk, NY, USA) at a significance level of *p* = 0.05.

## 5. Conclusions

We have validated animal research showing that miR-342-3p/-5p are anti-inflammatory/proangiogenic when significantly downregulated in T1DM, confirming an increased risk of CVD. Furthermore, as miR-342-3p/-5p were found to be markers of subclinical CVD, defining it at HbA_1c_ > 46.45 mmol/mol, (6.4%), they can be used for either the diagnosis or monitoring of subclinical CVD. Whilst miR-324-3p/-5p served as markers of vascular health and inflammation in all subjects, their beneficial effects require further research into pro-miR-342-3p/-5p as potential therapeutic interventions for T1DM patients.

## Figures and Tables

**Table 1:** Subjects’ clinical and metabolic characteristics.

Variable	Healthy Controls (*n* = 20)	Type 1 Diabetes Mellitus (*n* = 29)	*p*-Value
Age (years)	46.5 ± 11.7	47.2 ± 12.7	0.8
BMI (kg/m^2^)	26.0 ± 4.5	28.4 ± 6.7	0.3
Gender (M/F) n	9/11	14/15	-
HbA1c (mmol/mol)	35.1 ± 2.8	57.3 ± 7.6	<0.001 ***
HbA1c (%)	5.4 ± 0.3	7.4 ± 0.7	<0.001 ***
Glucose (mmol/L)	4.6 ± 1.0	9.7 ± 3.7	<0.001 ***
Hemoglobin (g/dL)	14.3 ± 1.2	14.5 ±1.2	1.0
Triglycerides (mmol/L)	1.4 ± 0.7	0.9 ± 0.4	0.008 **
Systolic Blood Pressure (mmHg)	117.5 ± 14.0	127.5 ± 9.4	0.005 **
CD45^dim^CD34^+^ CD133^+^ per 100 lymphocytes	0.009 ± 0.03	0.02 ± 0.01	<0.001 ***

** *p* < 0.01, *** *p* < 0.001.

## Abbreviations

cEPC	Circulating endothelial cell
CFU-Hill	Colony forming Unit-Hills colony
CVD	Cardiovascular disease
CXCR1/2	Chemokine receptor
FAA	Fibronectin Adhesion Assay
IKBKG	Inhibitor of nuclear factor kappa B kinase regulatory subunit Gamma
IPA	Ingenuity Pathway Analysis
MAP3K1	Mitogen-activated protein 3 kinase 1
PANX2	Pannexin-2
PBMCs	Peripheral blood mononuclear cells
SFTPA1	Surfactant protein A1
T1DM	Type 1 diabetes mellitus
TIMP-1	Tissue inhibitor of metalloproteases

## References

[1] Schram M.T., Chaturvedi N., Schalkwijk C.G., Fuller J.H., Stehouwer C.D., Group E.P.C.S. (2005). Markers of inflammation are cross-sectionally associated with microvascular complications and cardiovascular disease in type 1 diabetes--the EURODIAB Prospective Complications Study. Diabetologia.

[2] Livingstone S.J., Levin D., Looker H.C., Lindsay R.S., Wild S.H., Joss N., Leese G., Leslie P., McCrimmon R.J., Metcalfe W. (2015). Estimated life expectancy in a Scottish cohort with type 1 diabetes, 2008-2010. JAMA.

[3] Sibal L., Aldibbiat A., Agarwal S.C., Mitchell G., Oates C., Razvi S., Weaver J.U., Shaw J.A., Home P.D. (2009). Circulating endothelial progenitor cells, endothelial function, carotid intima-media thickness and circulating markers of endothelial dysfunction in people with type 1 diabetes without macrovascular disease or microalbuminuria. Diabetologia.

[4] Ahmed F.W., Rider R., Glanville M., Narayanan K., Razvi S., Weaver J.U. (2016). Metformin improves circulating endothelial cells and endothelial progenitor cells in type 1 diabetes: MERIT study. Cardiovasc. Diabetol..

[5] West D.J., Campbell M.D., Gonzalez J.T., Walker M., Stevenson E.J., Ahmed F.W., Wijaya S., Shaw J.A., Weaver J.U. (2015). The inflammation, vascular repair and injury responses to exercise in fit males with and without Type 1 diabetes: An observational study. Cardiovasc. Diabetol..

[6] Werner N., Kosiol S., Schiegl T., Ahlers P., Walenta K., Link A., Bohm M., Nickenig G. (2005). Circulating endothelial progenitor cells and cardiovascular outcomes. N. Engl. J. Med..

[7] Hill J.M., Zalos G., Halcox J.P., Schenke W.H., Waclawiw M.A., Quyyumi A.A., Finkel T. (2003). Circulating endothelial progenitor cells, vascular function, and cardiovascular risk. N. Engl. J. Med..

[8] Clark M., Kroger C.J., Tisch R.M. (2017). Type 1 Diabetes: A Chronic Anti-Self-Inflammatory Response. Front. Immunol..

[9] Hulsmans M., Holvoet P. (2013). MicroRNA-containing microvesicles regulating inflammation in association with atherosclerotic disease. Cardiovasc. Res..

[10] Schulte C., Barwari T., Joshi A., Theofilatos K., Zampetaki A., Barallobre-Barreiro J., Singh B., Sorensen N.A., Neumann J.T., Zeller T. (2019). Comparative Analysis of Circulating Noncoding RNAs Versus Protein Biomarkers in the Detection of Myocardial Injury. Circ. Res..

[11] Kantharidis P., Wang B., Carew R.M., Lan H.Y. (2011). Diabetes complications: The microRNA perspective. Diabetes.

[12] Assmann T.S., Recamonde-Mendoza M., De Souza B.M., Crispim D. (2017). MicroRNA expression profiles and type 1 diabetes mellitus: Systematic review and bioinformatic analysis. Endocr. Connect..

[13] Butz H., Kinga N., Racz K., Patocs A. (2016). Circulating miRNAs as biomarkers for endocrine disorders. J. Endocrinol. Investig..

[14] Simonson B., Das S. (2015). MicroRNA Therapeutics: The Next Magic Bullet?. Mini Rev. Med. Chem..

[15] Jung Y.Y., Kim K.C., Park M.H., Seo Y., Park H., Park M.H., Chang J., Hwang D.Y., Han S.B., Kim S. (2018). Atherosclerosis is exacerbated by chitinase-3-like-1 in amyloid precursor protein transgenic mice. Theranostics.

[16] Wang L., Xia J.W., Ke Z.P., Zhang B.H. (2019). Blockade of NEAT1 represses inflammation response and lipid uptake via modulating miR-342-3p in human macrophages THP-1 cells. J. Cell. Physiol..

[17] Lozano C., Estibals V., Duroux-Richard I., Apparailly F. (2019). miR-342-3p promotes cell survival and motility of osteoclast precursors. Ann. Rheum. Dis..

[18] Takahashi P., Xavier D.J., Evangelista A.F., Manoel-Caetano F.S., Macedo C., Collares C.V., Foss-Freitas M.C., Foss M.C., Rassi D.M., Donadi E.A. (2014). MicroRNA expression profiling and functional annotation analysis of their targets in patients with type 1 diabetes mellitus. Gene.

[19] Hezova R., Slaby O., Faltejskova P., Mikulkova Z., Buresova I., Raja K.R., Hodek J., Ovesna J., Michalek J. (2010). microRNA-342, microRNA-191 and microRNA-510 are differentially expressed in T regulatory cells of type 1 diabetic patients. Cell. Immunol..

[20] Chartoumpekis D.V., Zaravinos A., Ziros P.G., Iskrenova R.P., Psyrogiannis A.I., Kyriazopoulou V.E., Habeos I.G. (2012). Differential expression of microRNAs in adipose tissue after long-term high-fat diet-induced obesity in mice. PLoS ONE.

[21] Versari D., Daghini E., Virdis A., Ghiadoni L., Taddei S. (2009). Endothelial dysfunction as a target for prevention of cardiovascular disease. Diabetes Care.

[22] Calzascia T., Pellegrini M., Lin A., Garza K.M., Elford A.R., Shahinian A., Ohashi P.S., Mak T.W. (2008). CD4 T cells, lymphopenia, and IL-7 in a multistep pathway to autoimmunity. Proc. Natl. Acad. Sci. USA.

[23] Monti P., Bonifacio E. (2014). Interleukin-7 and type 1 diabetes. Curr. Diab. Rep..

[24] Cieri N., Camisa B., Cocchiarella F., Forcato M., Oliveira G., Provasi E., Bondanza A., Bordignon C., Peccatori J., Ciceri F. (2013). IL-7 and IL-15 instruct the generation of human memory stem T cells from naive precursors. Blood.

[25] Sudo T., Nishikawa S., Ohno N., Akiyama N., Tamakoshi M., Yoshida H., Nishikawa S. (1993). Expression and function of the interleukin 7 receptor in murine lymphocytes. Proc. Natl. Acad. Sci. USA.

[26] Lo H.C., Lin S.C., Wang Y.M. (2004). The relationship among serum cytokines, chemokine, nitric oxide, and leptin in children with type 1 diabetes mellitus. Clin. Biochem..

[27] Ha H., Debnath B., Neamati N. (2017). Role of the CXCL8-CXCR1/2 Axis in Cancer and Inflammatory Diseases. Theranostics.

[28] Williams R.O., Feldmann M., Maini R.N. (1992). Anti-tumor necrosis factor ameliorates joint disease in murine collagen-induced arthritis. Proc. Natl. Acad. Sci. USA.

[29] Ahmadi R., Heidarian E., Fadaei R., Moradi N., Malek M., Fallah S. (2018). miR-342-5p Expression Levels in Coronary Artery Disease Patients and its Association with Inflammatory Cytokines. Clin. Lab..

[30] Wei Y., Nazari-Jahantigh M., Chan L., Zhu M., Heyll K., Corbalan-Campos J., Hartmann P., Thiemann A., Weber C., Schober A. (2013). The microRNA-342-5p fosters inflammatory macrophage activation through an Akt1- and microRNA-155-dependent pathway during atherosclerosis. Circulation.

[31] Goon P.K., Lip G.Y., Boos C.J., Stonelake P.S., Blann A.D. (2006). Circulating endothelial cells, endothelial progenitor cells, and endothelial microparticles in cancer. Neoplasia.

[32] Avogaro A., Albiero M., Menegazzo L., de Kreutzenberg S., Fadini G.P. (2011). Endothelial dysfunction in diabetes: The role of reparatory mechanisms. Diabetes Care.

[33] McClung J.A., Naseer N., Saleem M., Rossi G.P., Weiss M.B., Abraham N.G., Kappas A. (2005). Circulating endothelial cells are elevated in patients with type 2 diabetes mellitus independently of HbA(1)c. Diabetologia.

[34] Werner N., Nickenig G. (2006). Influence of cardiovascular risk factors on endothelial progenitor cells: Limitations for therapy?. Arterioscler. Thromb. Vasc. Biol..

[35] Maxwell P.R., Timms P.M., Chandran S., Gordon D. (2001). Peripheral blood level alterations of TIMP-1, MMP-2 and MMP-9 in patients with type 1 diabetes. Diabet. Med..

[36] Ries C. (2014). Cytokine functions of TIMP-1. Cell. Mol. Life Sci..

[37] Cheng S., Cui Y., Fan L., Mu X., Hua Y. (2018). T2DM inhibition of endothelial miR-342-3p facilitates angiogenic dysfunction via repression of FGF11 signaling. Biochem. Biophys. Res. Commun..

[38] Colagiuri S., Lee C.M., Wong T.Y., Balkau B., Shaw J.E., Borch-Johnsen K., Group D.-C.W. (2011). Glycemic thresholds for diabetes-specific retinopathy: Implications for diagnostic criteria for diabetes. Diabetes Care.

[39] Expert Committee on Drug Dependence, Classification of Diabetes Mellitus (2003). Report of the expert committee on the diagnosis and classification of diabetes mellitus. Diabetes Care.

[40] D’Hondt C., Ponsaerts R., De Smedt H., Vinken M., De Vuyst E., De Bock M., Wang N., Rogiers V., Leybaert L., Himpens B. (2011). Pannexin channels in ATP release and beyond: An unexpected rendezvous at the endoplasmic reticulum. Cell. Signal..

[41] Makarenkova H.P., Shestopalov V.I. (2014). The role of pannexin hemichannels in inflammation and regeneration. Front. Physiol..

[42] Berchtold L.A., Miani M., Diep T.A., Madsen A.N., Cigliola V., Colli M., Krivokapic J.M., Pociot F., Eizirik D.L., Meda P. (2017). Pannexin-2-deficiency sensitizes pancreatic beta-cells to cytokine-induced apoptosis in vitro and impairs glucose tolerance in vivo. Mol. Cell. Endocrinol..

[43] Bond S.R., Naus C.C. (2014). The pannexins: Past and present. Front. Physiol..

[44] Kanter J.E., Bornfeldt K.E. (2013). Inflammation and diabetes-accelerated atherosclerosis: Myeloid cell mediators. Trends Endocrinol. Metab..

